# Studying Autism Using Untargeted Metabolomics in Newborn Screening Samples

**DOI:** 10.1007/s12031-020-01787-2

**Published:** 2021-01-30

**Authors:** Julie Courraud, Madeleine Ernst, Susan Svane Laursen, David M. Hougaard, Arieh S. Cohen

**Affiliations:** 1 Section for Clinical Mass Spectrometry, Danish Center for Neonatal Screening, Department of Congenital Disorders, Statens Serum Institut, Artillerivej 5, 2300 Copenhagen S, Denmark; 2 The Lundbeck Foundation Initiative for Integrative Psychiatric Research, iPSYCH, Fuglesangs Allé 26, 8210 Aarhus, Denmark

**Keywords:** Autism spectrum disorder, Dried blood spots, Untargeted metabolomics, Newborn screening, Biomarkers

## Abstract

Main risk factors of autism spectrum disorder (ASD) include both genetic and non-genetic factors, especially prenatal and perinatal events. Newborn screening dried blood spot (DBS) samples have great potential for the study of early biochemical markers of disease. To study DBS strengths and limitations in the context of ASD research, we analyzed the metabolomic profiles of newborns later diagnosed with ASD. We performed LC-MS/MS-based untargeted metabolomics on DBS from 37 case-control pairs randomly selected from the iPSYCH sample. After preprocessing using MZmine 2.41, metabolites were putatively annotated using mzCloud, GNPS feature-based molecular networking, and MolNetEnhancer. A total of 4360 mass spectral features were detected, of which 150 (113 unique) could be putatively annotated at a high confidence level. Chemical structure information at a broad level could be retrieved for 1009 metabolites, covering 31 chemical classes. Although no clear distinction between cases and controls was revealed, our method covered many metabolites previously associated with ASD, suggesting that biochemical markers of ASD are present at birth and may be monitored during newborn screening. Additionally, we observed that gestational age, age at sampling, and month of birth influence the metabolomic profiles of newborn DBS, which informs us on the important confounders to address in future studies.

## Introduction

It is estimated that autism spectrum disorder (ASD) affects more than 1% of all children worldwide (CDC 2019). ASD encompasses several neurodevelopmental disorders including autism, Asperger syndrome, pervasive developmental disorders, and childhood disintegrative disorder. The etiopathology of ASD is still unclear, and today, ASD is diagnosed based on behavioral signs and assessment of communication skills (World Health Organization 1993). How the condition should be classified is debated (Adam 2013). In this setting, early intervention is a challenge and has been reported to start in Europe at 42 months of age on average (Bejarano-Martín et al. 2019). Whether behavioral impairments are reflected in the blood as biochemical abnormalities is still unsure, but the quest for biomarkers is legitimate, as they would represent a useful tool to help in the diagnosis and treatment of ASD and in understanding its underlying molecular mechanisms (Shen et al. 2019).

The main risk factors for ASD include genetic (Bai et al. 2019) and non-genetic factors, especially exposure during fetal life (Newschaffer et al. 2007; Randall et al. 2018). Prenatal stress could influence fetal brain development and interact with genetic predispositions thereby enhancing the risk of future psychiatric disorders (Fine et al. 2014; Abbott et al. 2018). Among prenatal outcomes, maternal infection accompanied by fever during the second trimester of pregnancy has been found to increase the risk of ASD twofold approximately (Croen et al. 2019). Among perinatal outcomes, preterm birth (< 37 weeks) and low birth weight (small for gestational age) have been associated with an increased risk of ASD (Kuzniewicz et al. 2014).

Gastrointestinal tract disorders are often reported in ASD children, along with certain foods or diets impacting the severity of symptoms (Chaidez et al. 2014; Krajmalnik-Brown et al. 2015). There is a growing evidence of strong interactions between gut and brain through microbiota (Wang et al. 2018; Cerdó et al. 2019), and these observations support the notion that ASD is potentially connected to gut microbial populations and functions (Krajmalnik-Brown et al. 2015). It has also been shown that many small molecules differing between normally developing and ASD individuals likely result from microbial metabolism (De Angelis et al. 2015; Krajmalnik-Brown et al. 2015; Sharon et al. 2019). In humans, intestinal microbiota transplantation has shown very promising results, both against gastrointestinal tract symptoms and ASD symptoms, granting the therapy a ‘fast-track’ status by the FDA (Adams et al. 2019a). Among the plasma metabolites showing average to good classification capacity between the treated children and the controls, sarcosine, tyramine O-sulfate, and inosine 5′-monophosphate were selected as most discriminant (Adams et al. 2019b). Many of these studies postulate that microbiota-derived molecules are transported across the blood-brain barrier, acting as neuroactive metabolites (Cerdó et al. 2019). An impaired intestinal permeability or ‘leaky gut’ could also play a role in the effect of microbiota activity on psychiatric disorders (Magistris et al. 2010). If gut microbial metabolites of potential impact are indeed detectable in blood, this opens the door to blood-based investigations to further study and understand the metabolomic differences between ASD and non-ASD individuals in the context of gut-brain interactions.

Several studies have reported an altered metabolome associated with ASD during childhood, either in blood (Kang et al. 2018; Sharon et al. 2019; Barone et al. 2018; Anwar et al. 2018; Smith et al. 2019, 2020; West et al. 2014; Rangel-Huerta et al. 2019; Bitar et al. 2018; Delaye et al. 2018; Kuwabara et al. 2013), urine (Yap et al. 2010; Kałużna-Czaplińska 2011; Ming et al. 2012; Emond et al. 2013; Mavel et al. 2013; Noto et al. 2014; Gevi et al. 2016; Lussu et al. 2017; Anwar et al. 2018; Liu et al. 2019; Chen et al. 2019), or other matrices (Wang et al. 2019; Sharon et al. 2019). However, although some biochemical markers or set of markers seem promising (Shen et al. 2019), none has yet been proven robust enough for clinical practice. Furthermore, it remains unclear at what point in life biochemical abnormalities of ASD are detectable.

To study the early role of genetic, prenatal, and perinatal variables on disease development, samples need to be collected shortly after birth. However, it is not practically and ethically straightforward to draw blood from newborns prospectively. In many countries, the newborn screening programs are conducted on dried blood spots (DBS) collected a few days after birth. In Denmark, such DBS are stored in the Danish National Biobank and are available for research purposes for the last 40 years, thereby covering approximately half of the country’s population (Nørgaard-Pedersen and Hougaard 2007). This allows researchers to alleviate the biases inherent to recruitment in prospective clinical studies and instead retrospectively retrieve the samples that are connected to the relevant metadata stored in centralized health registries.

Taking advantage of this unique resource, we here aimed at studying the strengths and limitations of DBS samples in studying early biochemical abnormalities related to ASD development using an untargeted metabolomics protocol. We compared the metabolomic profiles of newborns that have been diagnosed with ASD by age 7 (cases) to newborns that have not (controls) and investigated potential main confounders. Although no clear case-control distinction was revealed, 18 compounds repeatedly reported in the ASD literature could be detected and three mass spectral features were differentially abundant in cases and controls before FDR correction. Additionally, we observed that gestational age, age at sampling, and month of birth influence the chemical profiles of neonates.

## Methods

### Materials

Methanol (MeOH), acetonitrile (ACN), isopropanol (IPA), water (H_2_O), and formic acid (FA) were of Optima™ LCMS-grade and were purchased from Thermo Fisher Scientific (Waltham, MA, USA). Stable-isotope-labeled internal standards (IS) from the NeoBase Non-derivatized MSMS kit (PerkinElmer, Waltham, MA, USA) were used. The exact list of compounds is provided in Online Resource 1.

### Subjects and Samples

Samples were drawn from children from the Integrative Psychiatric Research (iPSYCH) case-cohort sample (Pedersen et al. 2018). Aiming at studying genetic and environmental determinants of severe mental disorders, the iPSYCH sample has been selected from the entire Danish population born in 1981–2005. It comprises > 57,000 cases identified with ASD, schizophrenia, affective disorders, and/or attention-deficit/hyperactivity disorders and 30,000 controls (randomly sampled individuals). In our study, eligible cases were defined as born in 2005 and with a diagnosis of autism spectrum disorder (ICD10 F84.0, F84.1, F84.5, F84.8, and/or F84.9) (World Health Organization 1993) by the date of registry data extraction (2012). Out of these eligible cases, 37 children were randomly selected for the study. Children matching the cases’ gender and date of birth and without a diagnosis of psychiatric disorder were selected as eligible controls, of which one was randomly selected for each pair (37 controls). Other metadata, such as gestational age, birth weight, age at sampling, month of birth, mother’s age at birth, and date of diagnosis, were also collected from iPSYCH and the newborn screening database (when available).

Sample size was chosen for several reasons: (1) the unknown variation of metabolites in DBS made power calculations impossible, (2) batch effect is a common technical challenge in metabolomics, and analyzing all samples on one single 96-well plate was expected to reduce technical variability, and (3) DBS are highly precious samples.

DBS are whole blood from newborns, aged between 3 and 10 days (before 2009), blotted onto Ahlstrom #226 filter paper and left to dry for at least 3 h at room temperature before being sent by mail at ambient temperature to the Department of Congenital Disorders at the Statens Serum Institut in Copenhagen. Subsequent to being used in the newborn screening program, the samples are biobanked in the Danish National Biobank (www.nationalbiobank.dk) at − 20 °C until they are retrieved for further research analysis.

### Sample Extraction

A punch of 3.2-mm diameter was collected from each DBS using a Panthera-PuncherTM 9 blood spot punching system (PerkinElmer) directly into a MicroPlate, non-coated 96-well clear polystyrene plate (PerkinElmer). The IS were labeled amino acids (AA IS) and acylcarnitines (AC IS) diluted in 80% methanol (i.e., dilution factor of 1:330, concentrations in Online Resource 1) as extraction buffer. A total of 100 μL of extraction buffer was added to each well. The plate was heat-sealed and shaken for 45 min at 750 rpm at 25 °C in a PHMP-4 incubator. Then, it was centrifuged for 30 min at 4000 rpm at 4 °C.

All the transferring steps were performed on a Microlab STAR line automated liquid handling workstation using Venus software (Hamilton, Bonaduz, Switzerland). The supernatant (75 μL) was transferred to a hard-shell 96-well polypropylene PCR plate (Bio-Rad) and dried down with nitrogen 60 L/min at 25 °C for 1 h on an EVX-192 (Apricot Designs Evaporex). The residue was reconstituted in 75 μL 2.5% methanol, shaken for 15 min at 750 rpm at 25 °C in a PHMP-4 incubator, and centrifuged 10 min at 4000 rpm at 4 °C. A total of 65 µL was transferred to a hard-shell 96-well polypropylene PCR plate (Bio-Rad), heat-sealed, and centrifuged again for 5 min at 3000 rpm at 4 °C. The method from sample preparation to MS acquisition is also available as a table according to the guidelines for standardization of LCMS method reporting (Vogeser et al. 2019) with adaptation to metabolomics (Online Resource 1).

### Quality Assurance

LC-MS/MS instrument performance was controlled by analyzing four pooled extracts, eight solvent blanks, and three paper blanks at regular intervals. Pooled extracts were made of 5 µL of reconstituted extract from each of the samples (cases and controls only, total of 370 µL divided in fourwells spread across the plate) and were used to assess the consistency of extraction and data acquisition. Solvent blanks were used to check for carry over and instrument noise, while paper blanks (a punch of paper extracted like a sample) were used to monitor matrix signals from the paper. Internal standards were used to control the quality of the extraction, elution, and signal acquisition. Paired cases and controls were injected after one another but in a random order (first case, then control, or vice versa). Pairs were randomized over the plate.

### Liquid Chromatography

The samples were injected using an autosampler with stack cooler (Open Autosampler UltiMate OAS-3300TXRS, Thermo Fisher Scientific) and eluted through a Waters Acquity UPLC BEH C18 column (130 Å, 2.1 mm × 50 mm, 1.7-µm particles) preceded by a Waters Acquity UPLC BEH C18 VanGuard pre-column, 130 Å, 2.1 mm × 5 mm, 1.7-µm particles) using a Transcend II, LX-2 with UltiMate pumps (Thermo Fisher Scientific). The pressure limits were set at 0.0–1034.0 bar.

The mobile phase consisted of solvent A (97.31% H_2_O, 1.25% ACN, 1.25% MeOH, and 0.2% FA) and B (2.49% H_2_O, 48.66% ACN, 48.66% MeOH, and 0.2% FA). The Wash1 solvent was mobile phase A and the Wash2 solvent mix was 25:25:25:25 *v*/*v* MeOH/IPA/H_2_0/ACN + 0.2% FA. The gradient (0.25 mL/min) started with 100% A/0% B. After 0.5 min, we applied a gradient ramp to 0% A/100% B over 8.5 min followed by a 0.5-min flow ramp up to 0.9 mL/min and 5 min of 100% B. At 15 min, the column was equilibrated for 5.5 min with 100% A. At 17.5 min, the flow was changed back to 0.25 mL/min over 0.5 min. The total run time was 20.5 min, including 10-min sample run time and 10.5-min column wash and equilibration. The column temperature was maintained at 60.0 °C using a hot pocket column heater and the samples in the autosampler were kept at 4 °C throughout the analysis. The data was acquired in profile mode from 0.20 to 9.80 min.

### Mass Spectrometry

All samples were injected once and analyzed in data-dependent acquisition mode. The Q Exactive Orbitrap mass spectrometer (Thermo Fisher Scientific) was operated with a heated electrospray ionization source (HESI) in positive mode. The instruments were controlled using TraceFinder 4.1 Clinical Research and Aria MX (Thermo Fisher Scientific). Mass range in MS full scan mode was set to 70 to 1050 *m/z* with a resolution of 35,000. Automatic gain control was set to 1.10^6^, and maximum injection time at 100 ms. For data-dependent MS2, the resolution was set to 17,500. Automatic gain control was set to 1.10^5^ and maximum injection time at 50 ms. Loop count was 5, isolation window 1.5 *m/z*, and the stepped NCE 17.5, 35, and 52.5 eV. The spectrum data type was set to Profile. In data-dependent settings, the Apex trigger was set to 2 to 7 s with 15 s dynamic exclusion and charge exclusion on 3–8 and > 8. Diisooctyl phthalate (391.28429 *m/z*) was selected as lock mass. Other settings included the sheath gas pressure (N_2_, 32 psi), the auxiliary gas flow and temperature (N_2_, 8 arb. units, 350 °C), the S-lens radio frequency level (50.0%), the ion source temperature (350 °C), and the spray voltage (3.8 kV between 0–9.8 min and 1.0 kV between 9.8 and 10 min).

### LC-MS/MS Data Preprocessing

After conversion to .mzML (centroid) using MSConvertGUI v3.0 (ProteoWizard Software Foundation, Palo Alto, CA, USA) (Chambers et al. 2012), raw files were pre-processed using MZmine v2.41 (Katajamaa et al. 2006; Pluskal et al. 2010). All setting details are provided in the batch.xml file (Online Resource 2). Briefly, data were cropped based on retention time (RT) 0.27–9.80 min. Masses were detected with a noise threshold of 10,000 for MS1 and of 0 for MS2. The chromatogram was built using the ADAP module (Myers et al. 2017), with minimum seven scans per peak, a group intensity threshold of 10,000, a minimum highest intensity of 150,000, and a *m/z* tolerance of 0.001 *m/z* or 5 ppm. Deconvolution was performed using the Wavelets (ADAP) module, with *m/z* center calculation using median, and ranges for MS2 scan pairing of 0.01 Da and 0.4 min. The isotopes were grouped with a *m/z* tolerance of 0.001 *m/z* or 5 ppm and RT tolerance of 0.1 min. Peaks were aligned with a *m/z* tolerance of 0.001 *m/z* or 5 ppm and RT tolerance of 0.1 min, with 75% weight given to *m/z* and 25% to RT. Finally, peaks were filtered with a minimum of 15 peaks in a row, and the same RT and peak duration ranges as previously applied. The feature quantification table (.csv) and aggregated MS2 mass list (.mgf) were exported (no merging of MS/MS and filter rows: ALL) for further analysis.

### Feature-Based Molecular Networking Using GNPS and Compound Annotation

A molecular network was created with the feature-based molecular networking workflow (https://ccms-ucsd.github.io/GNPSDocumentation/featurebasedmolecularnetworking/) (Nothias et al. 2020) on the GNPS website (http://gnps.ucsd.edu) (Wang et al. 2016) by uploading the aggregated MS2 mass list. The data was filtered by removing all MS/MS fragment ions within ± 17 Da of the precursor *m/z*. MS/MS spectra were window filtered by choosing only the top six fragment ions in the ± 50 Da window throughout the spectrum. The precursor ion mass tolerance was set to 0.02 Da and a MS/MS fragment ion tolerance of 0.02 Da. A network was then created where edges were filtered to have a cosine score above 0.7 and more than 4 matched peaks. Further, edges between two nodes were kept in the network if and only if each of the nodes appeared in each other’s respective top 10 most similar nodes. Finally, the maximum size of a molecular family was set to 100, and the lowest scoring edges were removed from molecular families until the molecular family size was below this threshold. The spectra in the network were then searched against GNPS’ spectral libraries. The library spectra were filtered in the same manner as the input data. All matches kept between network spectra and library spectra were required to have a score above 0.7 and at least four matched peaks. The .graphml network file was then visualized using Cytoscape v3.7.2 (Shannon et al. 2003) where individual sample data and metadata were locally plotted (per sample and metadata sample group relative intensities). To enhance annotation of potential compounds of interest using the mzCloud spectral library (Thermo Fisher Scientific), .raw files were also preprocessed using Compound Discoverer 2.1 (CD2.1) SP1 software (Thermo Fisher Scientific). Details regarding the settings are provided in Online Resource 3. GNPS and Compound Discoverer (annotation reported when above mzCloud 80% confidence in identity or similarity search) offer annotations with a level 2 confidence according to the Metabolomics Standards Initiative (i.e., putative annotation) (Sumner et al. 2007; Schrimpe-Rutledge et al. 2016). To summarize and further enhance chemical structural information within the molecular network, substructure information (https://ccms-ucsd.github.io/GNPSDocumentation/ms2lda/) (Hooft et al. 2016), information from in silico structure annotations from Network Annotation Propagation (Silva et al. 2018) and Dereplicator (Mohimani et al. 2017) were incorporated using the GNPS MolNetEnhancer workflow (https://ccms-ucsd.github.io/GNPSDocumentation/molnetenhancer/) (Ernst et al. 2019) with chemical class annotations retrieved from the ClassyFire chemical ontology (Djoumbou Feunang et al. 2016). When no chemical structural information could be retrieved through the above searches, the MS/MS spectra were additionally searched via MASST (Wang et al. 2020) and SIRIUS + CSI:FingerID (Dührkop et al. 2015, 2019; Böcker and Dührkop 2016). MASST allows to query a single MS/MS spectrum across all public GNPS datasets giving an idea of the type of samples or matrices where the same MS/MS spectrum has been detected (Wang et al. 2020). SIRIUS + CSI:FingerID uses deep learning algorithms to predict the molecular and structural formula of a molecule from MS/MS spectra (Shen et al. 2014; Dührkop et al. 2015, 2019; Böcker and Dührkop 2016).

### Contamination Filtering and Further Data Curation

Using a Kendrick Mass Filter, we explored the compositionality of our data to assess the potential presence of undesired chemical background (da Silva et al. 2019). Out of the 4360 features obtained through MZmine preprocessing, more than 1100 possessed repeat units typical of polyethylene glycol (PEG) and polypropylene glycol (PPG). Filtering of PEG followed by filtering of PPG was performed using a Kendrick Mass Filter (da Silva et al. 2019) with the following parameters: number of observed signals = 5, Kendrick mass defect = 0.01, and fraction base = 1. All Jupyter notebooks used are publicly available on GitHub (https://github.com/SSI-Metabolomics/Autism_SupplementaryMaterial).

Of the 3253 remaining features, we further excluded those with a maximum intensity in paper blanks/maximum intensity in sample ratio ≥ 0.2, as well as features with 80% or more gaps (i.e., missing value) in cases and/or in controls (1975 features filtered).

### Data Visualization and Outlier Handling

To detect overall patterns in our data, we performed principal component analysis (PCA), which allowed us to assess the consistency of repeated pool injections (i.e., repeated injections of the same pooled samples should cluster in PCA). When performing these calculations on our “raw” unfiltered feature table (4360 features), seven samples were revealed as clear outliers, of which two controls and five cases. After contamination filtering and data curation (1281 features), six outliers remained since one outlier (control) was due to PEG contamination. Among the investigated potential explanations for these outliers, no pattern was found when looking at position on the plate layout, potential RT shift impairing the alignment, and metadata. However, targeted analysis of labeled internal standards and unlabeled endogenous homologs showed that significant (but unexplained) errors occurred during LC-MS/MS acquisition, with many undetectable compounds (TraceFinder 4.1 Clinical Research, Thermo Fischer Scientific) (Online Resource 4). A heatmap representation of the data (1,281 features) using MetaboAnalyst 4.0 (Chong et al. 2018) confirmed the six outliers with very low intensities (Online Resource 4). Therefore, we decided to exclude these outliers from further statistical analyses.

### Statistics

Using the filtered feature table, we calculated pair-wise Euclidean distances (1281 features, 68 samples) and performed permutational multivariate analyses of variance (PERMANOVAs) (Anderson 2001) to assess how much of the variance in the data is explained by a certain variable in the metadata. We investigated the following variables: ASD (yes/no), ASD subtype, gender, birth weight, gestational age, age at sampling, month of birth, and injection order. The Adonis *R*^2^ value indicates to what extent the variance is explained by the tested variable. Significance threshold was set at 0.05. Calculations were performed using the ggplot2, ggfortify, ggsci, rlang, viridis, and vegan packages in R software v4.0.3 (R Core Team 2020).

Finally, the curated dataset (1281 features, 68 samples, unpaired samples) was processed using MetaboAnalystR3.0 (Pang et al. 2020), R v4.0.3, and RStudio v1.3.1093. We replaced the remaining missing values by a small value (half the minimum positive value in the original data). We further applied a glog transformation and Pareto scaling. We ran a fold change analysis with a threshold of 2 (case/control or control/case). We performed *t* tests and Wilcoxon rank-sum tests with FDR correction for multiple comparisons. We performed PCA (including the four pooled samples). We could not reliably use the partial least squares discriminant analysis (negative Q^2^ in cross validation). All scripts used for statistical analysis are publicly available on GitHub (https://github.com/SSI-Metabolomics/Autism_SupplementaryMaterial).

## Results

### Subjects

Subjects’ characteristics are presented in Table 1 (details in Online Resource 5).

**Table 1:** Subjects characteristics

	*n* = 68			
	Cases		Controls	
Age at 1^st^ Jan. 2006 (median [range])	7.3 mo	[0.8–11.6]	7.5 mo	[0.8–11.6]
Gender (girls/boys)	7 / 25		8 / 28	
Classification of cases (ICD10)^a^				
- F84.0 Childhood autism	15		-	
- F84.1 Atypical autism	6		-	
- F84.5 Asperger syndrome	3		-	
- F84.8 Other pervasive developmental disorders	4		-	
- F84.9 Unspecified pervasive developmental disorders	10		-	
Gestational age (median [range])	40 weeks	[33–41]	39 weeks	[30–42]
Birth weight (median [range])	3498 g	[2210–4880]	3490 g	[977–4850]
Age at sampling (median [range])	6 days	[3–9]	6 days	[4–10]
Age of mother at birth (median [range])	32.3 years	[20.8–41.5]	31.8 years	[18.3–41.2]

More details are provided in Online Resource 5.

^a^ICD10 classification (World Health Organization 1993)

Cases and controls were similar in terms of gestational age, birth weight, age at sampling, and age of their mother at birth. The most prevalent ASD subtype was childhood autism. Most cases had only one diagnosis, but six had both unspecified pervasive development disorder and autism (either childhood autism or atypical autism). None had more than two diagnoses. Median age at first diagnosis was 5.6 years (range 1.1–7.8). Most subjects were born at term (gestational age ≥ 38 weeks). Only three cases and two controls were born preterm.

### Molecular Network Analysis

From all features for which a MS2 spectrum had been acquired (2217 features over 4360), a feature-based molecular network was computed via GNPS. Annotation could be retrieved for 150 (113 unique unlabeled) features (3.4%) of which 103 (83) by matching to GNPS libraries (annotation level 2) and 47 (30) by matching to our in-house library using Trace Finder (annotation level 1, Online Resource 6). Using the MolNetEnhancer workflow (Ernst et al. 2019), putative chemical structural information at the chemical class level, corresponding to a level 3 annotation, could be retrieved for an additional 859 features. Hence, nearly 46% (1009) of the mass spectral features could be putatively annotated at a level 1 to 3 (Online Resource 6). Annotation covered 31 chemical classes including 53 subclasses and 116 direct parents, such as medium-chain fatty acids, phosphatidylcholines, nucleotides, amino acids, bile acids, steroids, acylcarnitines, and catecholamines.

**Fig. 1:**
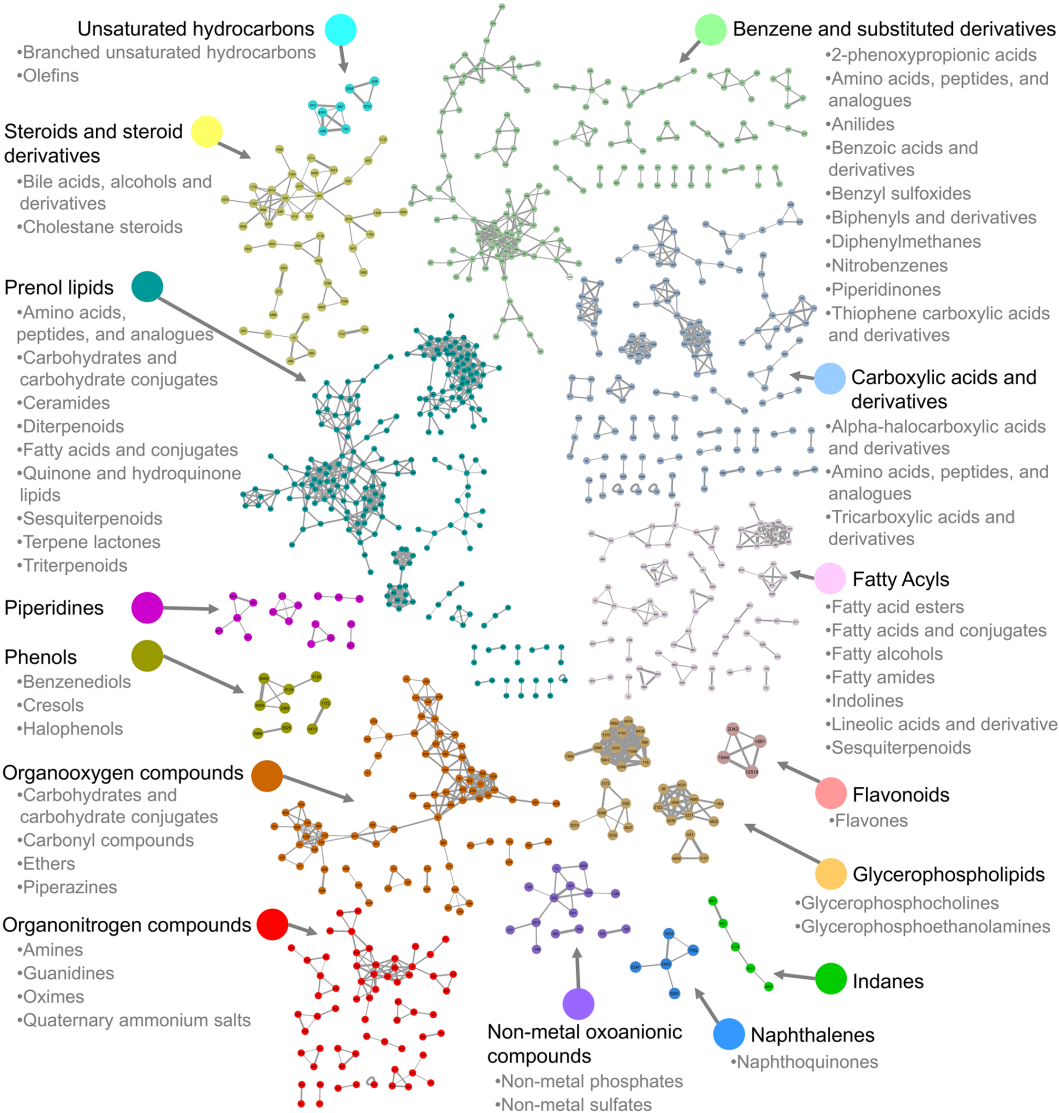
Feature-based molecular network displaying the 15 predominant putative chemical classes and their subclasses. Nodes represent mass spectral features and are used as a proxy for a metabolite. Connected nodes represent high tandem mass spectral similarity, and thus high chemical structural similarity. The thickness of the gray edges connecting nodes varies according to the cosine score representing to what extent two connected metabolites are chemically similar (based on MS2 spectra, from 0.7: less similar and thin edge to 1.0: identical and thick edge). The name of annotated metabolites (levels 1 and 2), details on chemical classes with fewer than 4 metabolites (absent on this figure), chemical classification scores (Ernst et al. 2019), all unknowns, and group intensities for all features (average, standard deviations) are detailed in Online Resource 6. Note that data represent a summary of most predominant classes per molecular family retrieved through either GNPS spectral library matching or in silico structure annotation and may contain false positives

**Fig. 2:**
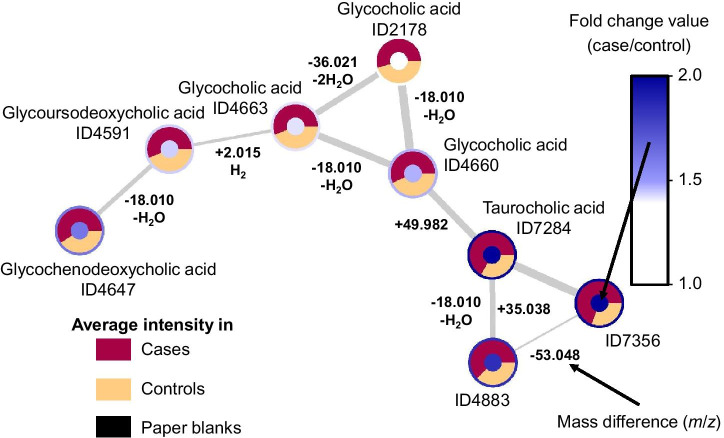
Network of molecular features putatively annotated as bile acids with average group intensities, fold change values, mass differences, and cosine scores displayed. Molecular family #75 is composed of eight bile acid structural analogues (see details in Online Resource 6). Coloring according to the fold change values makes it easier to spot the families with differential abundance in cases vs. controls. Displaying average intensities for the three groups (cases, controls, paper blanks) allows for a quick control of the matrix signals (paper blanks, here none of the features were detected in the matrix) and confirmation of fold change. On edges, while the thickness of the connection represents to what extent two metabolites are chemically similar, the mass difference is essential to support annotation as it translates into how molecules differ from one another (e.g., water loss, conjugation, adducts, etc.)

Molecular families (independent clusters of nodes) from the 15 predominant putatively annotated chemical classes are presented in Fig. 1 (see details in Online Resource 6). Plotting the average intensities in the three groups (cases, controls, paper blanks) in the ring of the nodes allowed to quickly spot clusters of features coming from noise or contaminants (detected in blanks) and focus on the others. To further ease the interpretation, we also plotted fold change values in the core of the nodes (one can also plot *P* values) to allow for a quick overview of the molecular families with potential biological relevance (see the example of bile acids in Fig. 2). This analysis showed the potential of DBS in covering various chemical classes and the power of feature-based molecular network analyses and related metabolome mining tools in expanding the interpretability of complex untargeted metabolomics data.

### Statistical Analyses

**Fig. 3:**
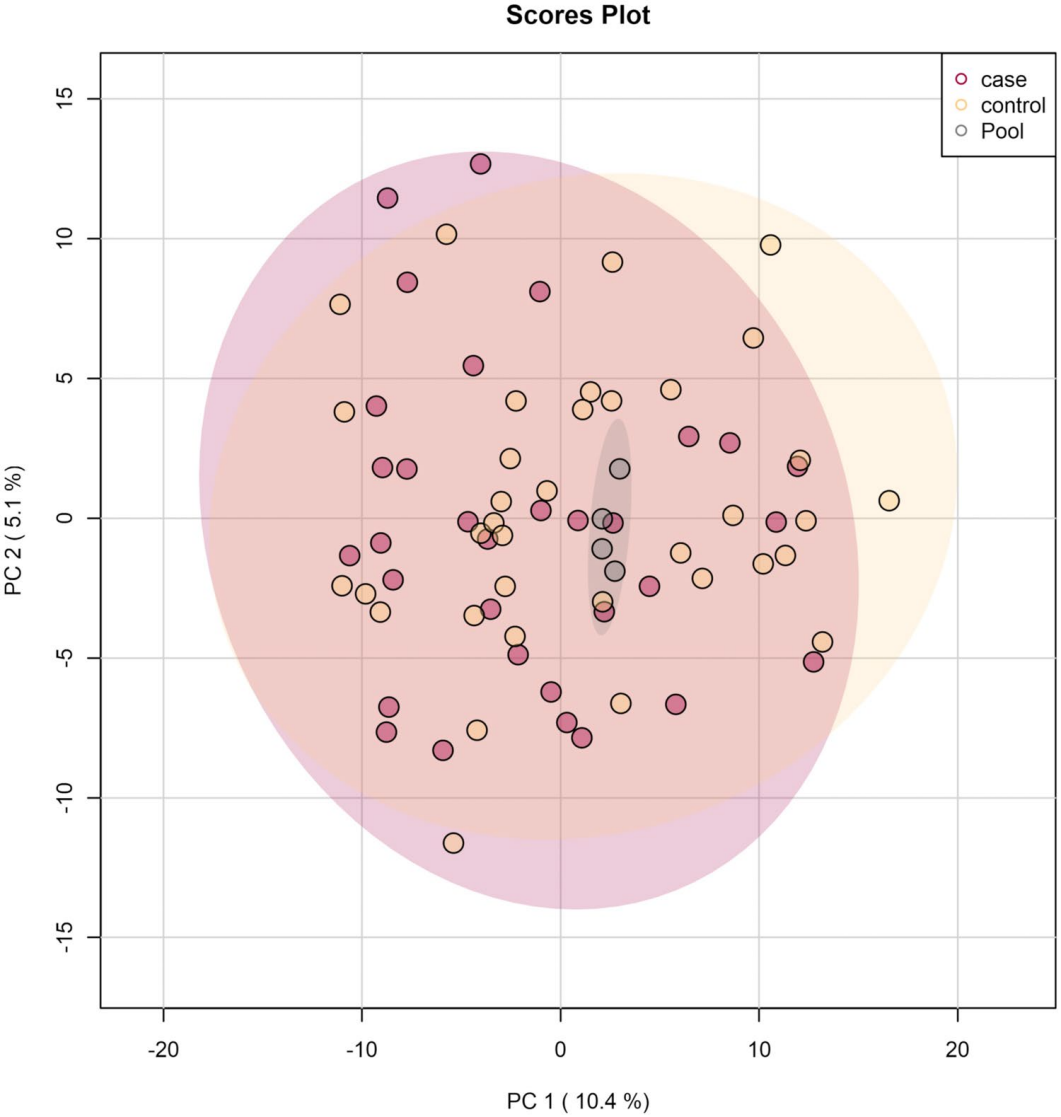
Principal component analysis of the 68 samples after outlier removal. Each sphere represents one sample. Axes are principal components 1 (x) and 2 (y) explaining 5.1% and 10.4% of the variation in the data, respectively. The four replicated pool injections cluster satisfactorily. Coloring reflects the type of samples, i.e., cases, controls and, four replicated pool injections. No clear distinction between cases and controls can be observed

Principal component analysis revealed that repeated pool injections clustered satisfactorily showing that the LC-MS/MS data acquisition was of acceptable quality (Fig. 3). When looking at the two groups (cases/controls), no clear separation was observed, even after removal of outliers (Fig. 3).

**Fig. 4:**
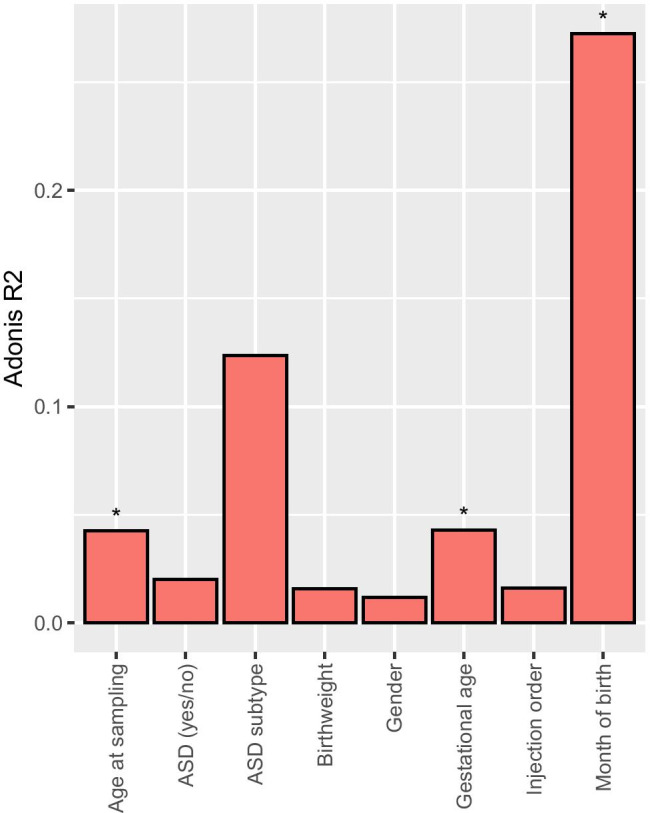
PERMANOVAs of the 68 samples after outlier removal showing how much of the variation (Adonis *R*^2^) is explained by a metadata variable. A star is present when the *P* values were < 0.05. All exact values are available in Online Resource 7 and detailed metadata (subject characteristics) are available in Table 1 and Online Resource 5

PERMANOVA (Fig. 4, Online Resource 7) revealed that the variance in the data was not significantly explained by the grouping (cases/controls) (Adonis *R*^2^ = 0.0199, *P* value = 0.226), even when distinguishing subtypes of ASD (Adonis *R*^2^ = 0.123, *P* value = 0.546, see Table 1 for details on subtypes of ASD). Similarly, the gender, birth weight, and injection order did not significantly explain the variance in the data (Adonis *R*^2^ < 0.02, *P* value > 0.05). However, variation in the data explained by gestational age (Adonis *R*^2^ = 0.0429, *P* value = 0.021), age at sampling (Adonis *R*^2^ = 0.0425, *P* value = 0.016) and especially month of birth (Adonis *R*^2^ = 0.272, *P* value = 0.001) was significant (Fig. 4).

Results of univariate analyses and fold change analysis were carefully scrutinized feature by feature. Considering our small sample size and potential pitfalls inherent to untargeted metabolomics related to contaminants or integration errors, we thought essential to inspect each result to eliminate false positives and spurious findings. Our inspection consisted of a five-step logic starting with peak integration and shape quality (MZmine). We then plotted all individual intensity values to assess whether the case/control difference was driven by four or fewer samples. If not, we reported the extent of missing values in each group, checked the consistency of replicated pool injections, and finally checked whether the feature was present in the feature-based molecular network, annotated as a contaminant or in a node cluster with such annotation (Online Resource 8). A large proportion of the inspected features were excluded based on these criteria, showing the importance of such a verification in order not to pursue spurious findings in future studies.

Among the 24 features with a fold change (case/control) value < 0.5 or > 2.0, only one passed manual inspection (Table 2, the full table is in Online Resource 8).

**Table 2:** Differentially abundant features in univariate analyses without FDR correction (*p* < 0.01, two features) and/or with high fold change (one feature) meeting inspection criteria

Putative annotation of relevant compounds	Annotation level^a^	m/z	RT (min)	ID	*p* value *t* test		*p* value Wilcoxon rank-sum test		FC^b^	Network connections^c^
					without FDR correction	with FDR correction	without FDR correction	with FDR correction		
Methacholine C_8_H_18_NO_2_^+^	2^**d**^	160.13315	0.45	159	0.0021	0.9174	0.0031	0.9434	1.25	Connected to 1853
SIRIUS 99.96%: C_11_H_22_N_2_O_3_ (M + H +)	4	231.17005	2.78	5593	0.0072	0.9174	0.0138	0.9434	1.46	Single node
SIRIUS 7.12%: C_36_H_63_N_21_O_14_ (M + H +)	4	1014.48923	6.64	8605	0.0414	0.9174	0.0179	0.9434	0.42	Single node

^a^Annotation level of confidence according to the Metabolomics Standards Initiative (i.e., putative annotation) (Sumner et al. 2007; Schrimpe-Rutledge et al. 2016)

^b^FC: Fold change (case/control)

^c^Network connections in GNPS feature-based molecular network

^d^Source of annotation mzCloud (89.9% score). See its mass spectrum in Online Resource 9

Inspection criteria: peak integration or shape quality, initial missing values, single values plot, presence, and consistence in replicated pool injections, annotation, or connection to contaminants. For details, see Online Resource 6.

Eluting quite late (RT = 6.64 min, ID8605), this relatively hydrophobic compound had a detected *m/z* of 1014.4892 and was not connected to any other node in the network analysis (see its mass spectrum in Online Resource 9). It could not be annotated, but the algorithm of SIRIUS + CSI:FingerID pointed at a raw formula of C_36_H_63_N_21_O_14_ ([M + H] + , only 7.12% scoring). This compound was more than twice as intense in controls as in cases (FC 0.42, average intensity in cases 2.73E + 05 and controls 7.51E + 05) and would need further investigation, especially as it was not detected in many samples (Online Resource 8). A MASST search was performed; however, the feature with *m/z* 1014.4892 was not found in any of the public datasets on GNPS.

No feature was significantly differentially abundant in cases and controls according to the univariate analyses with FDR correction for multiple comparisons (*P* values in Table 2).

Features that were differentially abundant before FDR correction are presented in Table 2. As a high proportion of features were deemed irrelevant after inspection, we are presenting only the two relevant features that passed our quality control criteria. The full list and inspection details can be found in Online Resource 8. Methacholine was found to be significantly more abundant in cases when compared with controls (average intensity in cases 4.41E + 07 and controls 3.94E + 07) both when using a *t* test (*p* = 0.0021) and a Wilcoxon rank-sum test (*p* = 0.0031). The corresponding node (ID159) in the network analysis was connected to another node with a mass difference of − 0.036 *m/z* (225 ppm) which could not be annotated. None of the applied metabolome mining tools was able to retrieve chemical structural information for the second compound significantly more abundant in cases than in controls (ID5593, *m/z* 1014.4892, average intensity in cases 5.71E + 05 and controls 4.35E + 05). SIRIUS + CSI:Finger ID predicted a molecular formula of C_11_H_22_N_2_O_3_ (M+H+, 99.96% scoring). Its RT of 2.78 min could indicate a medium polarity with a logP between − 1.0 and 0.5 when compared with tryptophan (RT 2.56 min, HMDB experimental logP − 1.06) and hippuric acid (RT 3.04 min, HMDB experimental logP 0.31).

Among the 273 compounds reported in two recent reviews (Glinton and Elsea 2019; Shen et al. 2019), 22 were cited at least three times in ASD literature, of which 18 could be linked to features in our study after manual verification (Table 3, Online Resource 10).

**Table 3:** Compounds reported in the literature three or more times as being associated with ASD

Compound name & HMDB ID	Annotation level^a^	Raw formula	*m/z* [M + H]+	RT (min)	Feature ID (MZmine 2.41)	Detected by Compound Discoverer 2.1	Literature reference
Arginine	1	C_6_H_14_N_4_O_2_	175.11895	0.35	1450	ND	(Kuwabara et al. 2013; Anwar et al. 2018; Liu et al. 2019)
HMDB0000517							
Aspartic acid	1	C_4_H_7_NO_4_	134.04478	0.41	1073	ND	(De Angelis et al. 2013; West et al. 2014; Kang et al. 2018; Liu et al. 2019; Wang et al. 2019; Rangel-Huerta et al. 2019)
HMDB0000191							
Citric acid	4	C_6_H_8_O_7_	193.03428	0.35	1776	Yes	(Kałużna-Czaplińska 2011; West et al. 2014; Bitar et al. 2018)
HMDB0000094							
Creatine	2	C_4_H_9_N_3_O_2_	132.07675	0.40	16	Yes	(Mavel et al. 2013; Lussu et al. 2017; Bitar et al. 2018)
HMDB0000064							
Creatinine	2	C_4_H_7_N_3_O	114.06619	0.40	281	Yes	(West et al. 2014; Lussu et al. 2017; Liu et al. 2019; Chen et al. 2019)
HMDB0000562							
Decanoylcarnitine	1	C_17_H_33_NO_4_	316.24823	6.00	3633	Yes	(Barone et al. 2018; Wang et al. 2019; Rangel-Huerta et al. 2019)
HMDB0000651							
Glutamic acid	1	C_5_H_9_NO_4_	148.06043	0.38	136	Yes	(De Angelis et al. 2013; West et al. 2014; Lussu et al. 2017; Kang et al. 2018; Anwar et al. 2018; Bitar et al. 2018; Delaye et al. 2018; Rangel-Huerta et al. 2019)
HMDB0000148							
Glutamine	2	C_5_H_10_N_2_O_3_	147.07642	0.40	107	Yes	(Kang et al. 2018; Anwar et al. 2018; Smith et al. 2019)
HMDB0000641							
Glycine	3	C_2_H_5_NO_2_	76.03930	0.38	1177	ND	(Ming et al. 2012; Mavel et al. 2013; De Angelis et al. 2013; Lussu et al. 2017; Delaye et al. 2018; Smith et al. 2019, 2020)
HMDB0000123							
Glycolic acid	-	C_2_H_4_O_3_	77.02332	-	ND	ND	(Emond et al. 2013; Noto et al. 2014; Chen et al. 2019)
HMDB0000115							
Hippuric acid	2	C_9_H_9_NO_3_	180.06552	3.04	5174	ND	(Yap et al. 2010; Kałużna-Czaplińska 2011; Emond et al. 2013; Lussu et al. 2017)
HMDB0000714							
Histidine	2	C_6_H_9_N_3_O_2_	156.07675	0.32	342	Yes	(Ming et al. 2012; De Angelis et al. 2013; Gevi et al. 2016)
HMDB0000177							
Lactic acid	-	C_3_H_6_O_3_	91.03897	-	ND	ND	(Kuwabara et al. 2013; Lussu et al. 2017; Kang et al. 2018; Smith et al. 2020)
HMDB0000190							
p-cresol	-	C_7_H_8_O	109.06479	-	ND	ND	(De Angelis et al. 2013; Gevi et al. 2016; Kang et al. 2018; Chen et al. 2019)
HMDB0001858							
Phenylalanine	1	C_9_H_11_NO_2_	166.08625	1.70	594 + 5370 + 287	Yes	(De Angelis et al. 2013; Gevi et al. 2016; Wang et al. 2019)
HMDB0000159							
Serine	2	C_3_H_7_NO_3_	106.04987	0.40	437	ND	(Ming et al. 2012; De Angelis et al. 2013; West et al. 2014; Bitar et al. 2018; Delaye et al. 2018)
HMDB0000187							
Succinic acid	-	C_4_H_6_O_4_	119.03388	-	ND	ND	(Yap et al. 2010; Emond et al. 2013; Mavel et al. 2013; West et al. 2014; Smith et al. 2020)
HMDB0000254							
Taurine	3	C_2_H_7_NO_3_S	126.02194	0.43	428	ND	(Yap et al. 2010; Ming et al. 2012; Kuwabara et al. 2013; Mavel et al. 2013; Lussu et al. 2017; Sharon et al. 2019)
HMDB0000251							
Threonine	2	C_4_H_9_NO_3_	120.06552	0.40	476	ND	(Ming et al. 2012; Anwar et al. 2018; Bitar et al. 2018; Liu et al. 2019)
HMDB0000167							
Tryptophan	2	C_11_H_12_N_2_O_2_	205.09715	2.53	164	Yes	(Noto et al. 2014; Gevi et al. 2016; Lussu et al. 2017; Anwar et al. 2018; Rangel-Huerta et al. 2019)
HMDB0000929							
Tyrosine	1	C_9_H_11_NO_3_	182.08117	0.72	58	Yes	(Kang et al. 2018; Bitar et al. 2018; Wang et al. 2019)
HMDB0000158							
Valine	2	C_5_H_11_NO_2_	118.08625	0.42	ND	Yes	(De Angelis et al. 2013; Lussu et al. 2017; Smith et al. 2019)
HMDB0000883							

*ND* not detected

^a^Annotation level of confidence according to the Metabolomics Standards Initiative (i.e., putative annotation) (Sumner et al. 2007; Schrimpe-Rutledge et al. 2016)

When the [M + H]+ adduct could not be found (± 5 ppm), common adducts were searched including [M + Na]+, [M + K]+, [M + 2H]2+, and [M + H-H_2_O]+.

See full list of compounds considered and more details in Online Resource 10.

### Discussion

To assess the potential of newborn DBS to study early biochemical markers of ASD shortly after birth, we compared DBS samples from newborns that have later on been diagnosed with ASD to newborns that have not. Our study showed the capacity of untargeted metabolomics as an analytical tool applied to biobanked DBS samples to cover several metabolites relevant to ASD, thus suggesting that biochemical markers of ASD are present at birth and could be targeted during neonatal screening. In addition, our method pinpointed other factors which have a strong influence on the metabolic profile of newborn DBS, such as gestational age, age at sampling and month of birth, and which are important to consider when designing metabolomic studies in neonatal, biobanked DBS.

One study from 2013 was performed on newborn DBS samples from 16 autistic children and assessed 90 biomarkers (not only small molecules) using immunoassays (Mizejewski et al. 2013) of which three sets of five were associated with ASD. Another study was performed on DBS but in older ASD children (*n* = 83, age 2–10 years) and was targeting 45 metabolites (Barone et al. 2018), of which 9 were significantly higher in ASD children. However, the potential of DBS in untargeted metabolomics studies has not yet been fully studied, and never in the context of ASD (see recent reviews (Glinton and Elsea 2019; Shen et al. 2019)).

Among the 22 compounds that had been repeatedly (≥ 3 times) reported in the literature to be involved in ASD, 18 could be putatively annotated in our study, showing that our analytical pipeline covers many relevant metabolites, including some specific to gut microbiota activity. Despite thorough curation and inspection of the acquired data, no feature was significantly differentially abundant in cases and controls after FDR correction. This shows that a bigger sample size will be required for the study of ASD using newborn DBS along with appropriate consideration of the confounders specific to these samples to reduce their impact.

Among the hits and interesting findings of our study, we could show that a methacholine structural analog could be a relevant marker for ASD, as it was found at a higher—although not significant—abundance in newborns that have been diagnosed with ASD by age 7. Methacholine is a choline ester drug acting as non-selective muscarinic receptor agonist. It is mainly known as methacholine chloride for its use in assessing bronchial hyper-reactivity in asthmatic patients. Although muscarinic receptors were not associated with ASD in children (Lee et al. 2002), lower estimates of ASD risk among children exposed during fetal life to muscarinic receptor 2 agonists were reported (Janecka et al. 2018). Higher abundance of methacholine in DBS of ASD cases, as seen in our study, would therefore not be easily explained and demand further investigation. However, detecting a drug metabolite such as methacholine in newborn samples is unexpected; thus, it is more likely that this feature is an endogenous choline ester with similar fragmentation behavior to methacholine.

Two other unknown features would benefit from being monitored in future studies. One relatively hydrophobic compound (ID8605, *m/z* 1014.4892) showed an important fold change (much lower in cases) but was not detected in many samples maybe due to low intensities. The second compound (moderately polar, ID5593, *m/z* 1014.4892, C_11_H_22_N_2_O_3_) was significantly higher in cases before FDR correction and detected in more than 65% of samples.

We have shown that gestational age, age at sampling, and month of birth are strong drivers of metabolomic profiles in newborn DBS samples. This demonstrates the importance of considering these confounders when designing a future study using such samples.

Prematurity has been involved in numerous adverse health outcomes (Saigal and Doyle 2008) and metabolic maturity has previously been shown to be reflected in the blood and other matrices of infants after birth (Gil and Duarte 2018; Ernst et al. 2020). Although, in the present study, only three cases and two controls were premature (< 38 weeks of gestational age), we saw a significant effect of gestational age on the metabolomic profile of newborns thus showing that gestational age is an important factor to be controlled for in newborn DBS studies.

Similarly, we found that age at sampling has a significant impact on the newborn blood metabolome. From 3 to 10 days of age, only 1 week has passed, and yet fundamental metabolic changes occur in the newborn possibly in connection with post-natal nutrition, the maturation of the newborn’s microbiome as well as environmental conditions (healthcare, hospital vs home, etc.). The endogenous anabolism/catabolism balance is in itself a strong variable to consider at that age. From 2009 onwards, the Danish newborn screening program has indeed chosen to standardize the age at DBS sampling to 48 to 72 h to optimize the window where potential inborn errors of metabolism would be detected best and as early as possible since quick intervention is essential in such cases (Dionisi-Vici et al. 2006). The iPSYCH sample was based on diagnoses of psychiatric disorders recorded in Danish health registries in 2012 (Pedersen et al. 2018). Such diagnoses are often given after several years of age, which is why the iPSYCH sample included subjects born latest in 2005, year at which the age at sampling was not so narrowly standardized.

Another major change that occurs in newborns at birth and in the following days is the gut maturation and its further colonization by microbes (Milani et al. 2017). This topic has been under expanding attention in the last decade, and the development and involvement of gut microbiota in neurodevelopment is being scrutinized extensively (Cerdó et al. 2019). The exact dynamics of the microbiota development in the placenta and during the first days of life is still uncertain (Backhed et al. 2015; Milani et al. 2017), as well as to what extent its activity can be reflected in the blood. A recent study has shown that gut microbial alpha-diversity can be predicted from the human blood metabolome (Wilmanski et al. 2019) suggesting that microbial metabolites explain a significant amount of the variation in the human blood metabolome. Thus, although sampled at an early stage in life, it is plausible that microbial metabolites mediating health may be found in dried blood spots from newborns (Ernst et al. 2020). Studying both fecal and blood samples will be essential to answer questions related to the impact of gut microbes on the gut-brain axis, especially in the context of psychiatric disorders where the brain is the main organ concerned but indeed located quite far from the gut. Microbial metabolites would necessarily need to travel in the blood (or lymph) and through the blood-brain barrier to interact with the brain. In our study, some detected metabolites could partly derive from gut microbiota activity such as DL-indole-3-lactic acid (ID3461, Meng et al. 2020; Laursen et al. 2020), taurine (ID428, level 3, Sharon et al. 2019), various bile acids (Online Resource 6, Wang et al. 2019), or inosine 5′-monophosphate (ID1133, level 3, Adams et al. 2019b).

Lastly, we found that month of birth explains a significant variation in metabolomic profiles of newborns (Fig. 4). Whether there is a yearly cyclic pattern or whether our findings are specific to 2005 remains to be determined. Explanations could include aspects related to pregnancy conditions varying along the year such as diet, weather conditions and sun exposure (e.g., impact on vitamin D levels, type and extent of physical and social activities, mood and stress (Keller et al. 2005)), exposure to “seasonal” infectious diseases (e.g., influenza), exposure to varying air quality (e.g., pollution or pollens (D’Amato et al. 2015)), as well as sample storing conditions which might fluctuate over the year (e.g., sample transport at higher temperatures during summer).

Gender and birth weight were not found to explain a significant part of the variance in the metabolomic profiles of newborn DBS samples in our study, despite the obvious connection between gestational age and birth weight. The gender misbalance which reflects the gender disparity in ASD (a quarter were girls) and small sample size could explain this finding. Some studies have indeed reported that the profile of newborn girls and boys differed in, for instance, blood amino acids and acylcarnitines (Ruoppolo et al. 2015), as well as urine profiles (Diaz et al. 2016). Despite our finding, we believe that gender and birth weight should be adjusted for and taken into consideration when designing metabolomics studies in newborns. Several of the tested confounders are inter-connected with, for instance, reports of more males being born preterm (Challis et al. 2013) and females being born lighter (Wilkin and Murphy 2006), both associations being explained by mechanisms that are likely to be reflected in the metabolome such as inflammatory response and insulin resistance, respectively.

### Limitations and Strengths

To minimize the use of highly valuable and rare samples, we analyzed only 37 pairs of cases and controls in this study aiming at assessing the potential of DBS samples in ASD research. Despite the small sample size that did not confer enough statistical power for pinpointing strong marker metabolites of ASD, we could detect numerous metabolites associated with ASD in previous studies and identify a number of confounders to be considered in future untargeted metabolomics study using newborn DBS. Other confounders not evaluated in our study will need to be assessed in future studies, including metabolic changes in DBS associated with time and storage conditions. Hematocrit variation could not be measured in our study as we had access to only one punch of paper and did not have the possibility to measure a surrogate marker such as potassium in the same punch as done by others (Petrick et al. 2017). Furthermore, metabolites detected in this study are inherently reflective of sampling protocols, including extraction protocols and MS acquisition parameters, and should be interpreted within these limitations.

## Conclusions

This is the first untargeted metabolomics study assessing the potential of biobanked newborn DBS samples in ASD research. The development of biobanks and reuse of systematically collected DBS samples for research purposes in connection with registry data represent many new opportunities to study the physiopathology and early signs of diseases, with extraordinary impacts in prevention, diagnosis, and treatment strategies. We showed that untargeted metabolomics on DBS samples offer a wide and relevant coverage of metabolites for the study of ASD and that the new processing tools used in our method largely expand the interpretability of such complex data.

## Supplementary Information

Below is the link to the electronic supplementary material.

Supplementary file1: Online Resource 1: Standardized reporting of untargeted metabolomics LC–MS/MS method according to (Vogeser et al. 2019) containing the list of internal standards from the Neobase Non-derivatized MSMS kit and their concentration in the extraction buffer. (XLSX 20 KB)

Supplementary file2: Online Resource 2: MZmine batch.xml file used to preprocess the raw data. (XML 11 KB)

Supplementary file3: Online Resource 3: Compound Discoverer 2.1 preprocessing workflow settings. (PDF 245 KB)

Supplementary file4: Online Resource 4: Targeted analysis of outliers using TraceFinder (IS and unlabeled homologs) and heatmap of untargeted analysis. (PDF 599 KB)

Supplementary file5: Online Resource 5: Subjects characteristics in details. (XLSX 14 KB)

Supplementary file6: Online Resource 6: All features including annotated compounds. Out of the 4360 features detected, 150 (113 unique unlabeled) could be annotated by GNPS library matching (annotation level 2) or in-house TraceFinder library (annotation level 1) and an additional 859 by MolNetEnhancer (annotation level 3). (XLSX 1145 KB)

Supplementary file7: Online Resource 7: PERMANOVAs Adonis R2 values and p-values calculated without outliers (68 samples). (XLSX 11 KB)

Supplementary file8: Online Resource 7: PERMANOVAs Adonis R2 values and p-values calculated without outliers (68 samples). (XLSX 37 KB)

Supplementary file9: Online Resource 9: Fragmentation profiles of the two unknown features to be monitored in future studies as well as methacholine as shown in Table 2 (PDF 174 KB)

Supplementary file10: Online Resource 10: Full list of compounds reported in the literature as involved in ASD and considered in this study. (XLSX 44 KB)

## Data Availability

The datasets generated and/or analyzed during the current study are not publicly available due to the risk of compromising individual privacy but are available from the corresponding author on reasonable request and provided that an appropriate collaboration agreement can be agreed upon.

## Code availability

All scripts used for data processing and statistical analyses are publicly available on GitHub (https://github.com/SSI-Metabolomics/Autism_SupplementaryMaterial).
