# Supplementary material for: Studying Autism Using Untargeted Metabolomics in Newborn Screening Samples
Source: J Mol Neurosci. 2021 Jan 30;71(7):1378–93. doi: 10.1007/s12031-020-01787-2 (PMC8233278; doi:10.1007/s12031-020-01787-2)

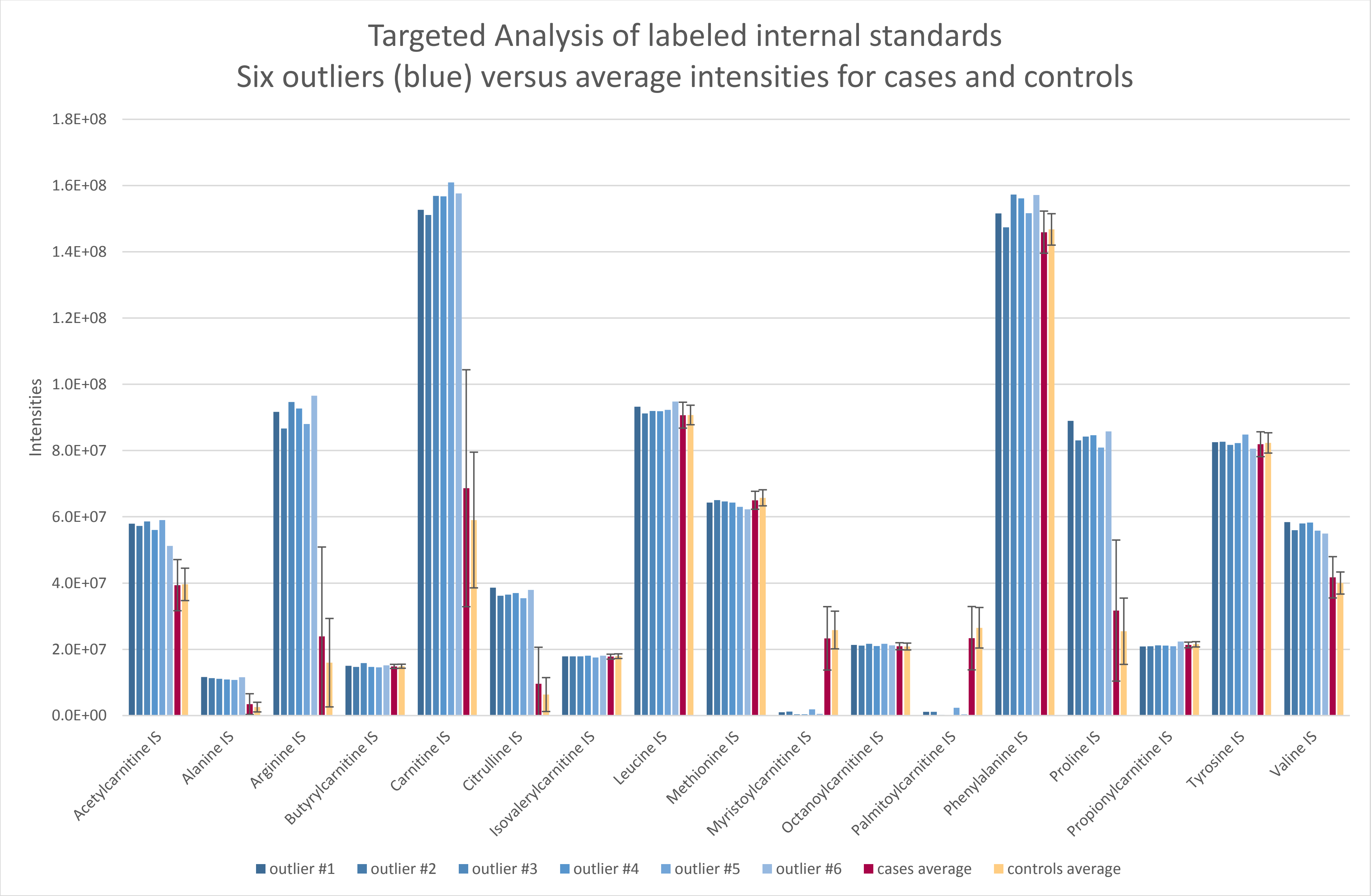

Targeted Analysis of labeled internal standards
Six outliers (blue) versus average intensities for cases and controls
Intensities
1.8E+08
1.6E+08
1.4E+08
1.2E+08
1.0E+08
8.0E+07
6.0E+07
4.0E+07
2.0E+07
0.0E+00
Acetylcarnitine IS
Alanine IS
Arginine IS
Butyrylcarnitine IS
Carnitine IS
Citrulline IS
Isovalerylcarnitine IS
Leucine IS
Methionine IS
Myristoylcarnitine IS
Octanoylcarnitine IS
Palmitoylcarnitine IS
Phenylalanine IS
Proline IS
Propionylcarnitine IS
Tyrosine IS
Valine IS
outlier #1
outlier #2
outlier #3
outlier #4
outlier #5
outlier #6
cases average
controls average

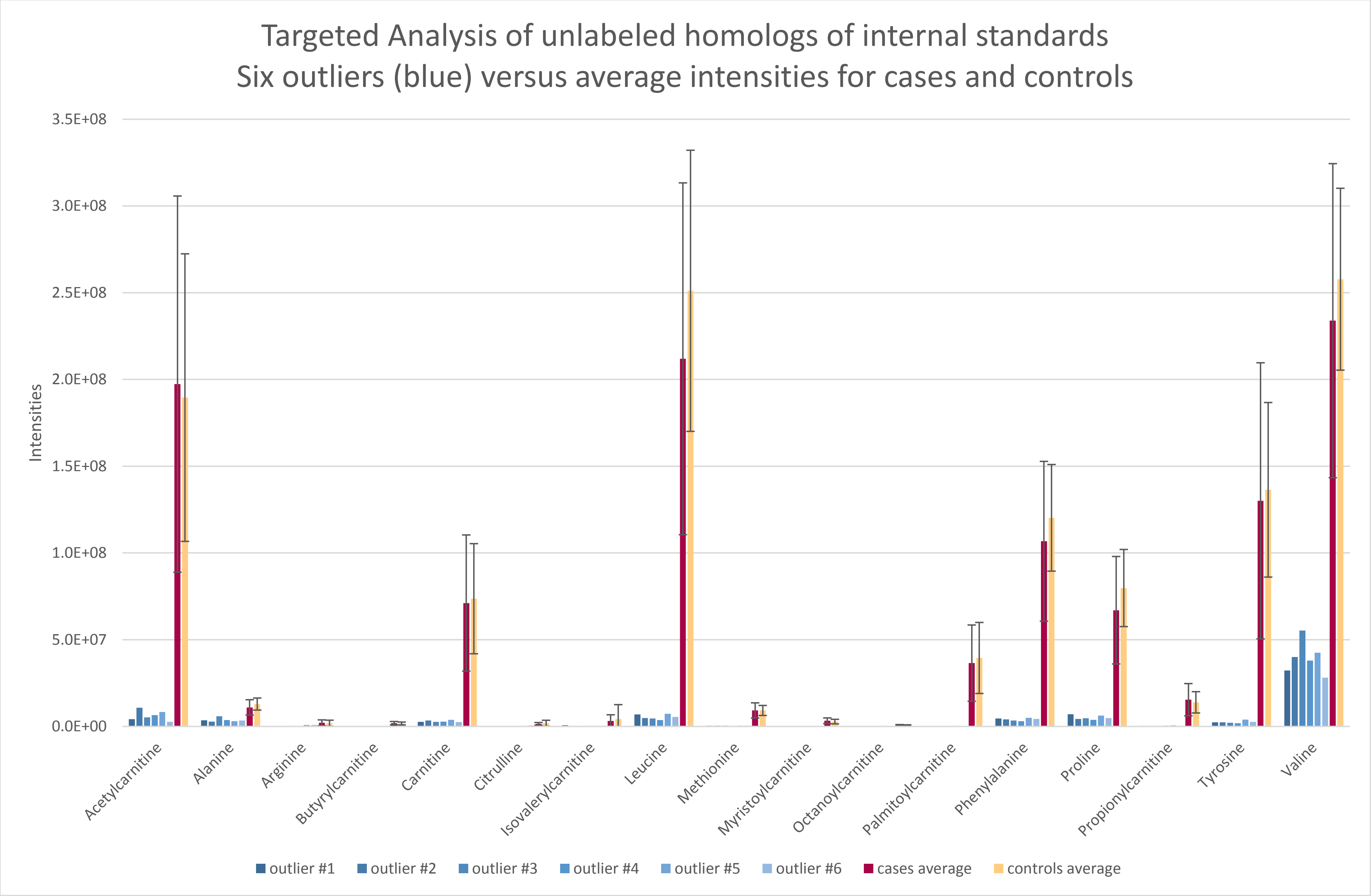

Targeted Analysis of unlabeled homologs of internal standards
Six outliers (blue) versus average intensities for cases and controls
Intensities
3.5E+08
3.0E+08
2.5E+08
2.0E+08
1.5E+08
1.0E+08
5.0E+07
0.0E+00
Acetylcarnitine
Alanine
Arginine
Butyrylcarnitine
Carnitine
Citrulline
Isovalerylcarnitine
Leucine
Methionine
Myristoylcarnitine
Octanoylcarnitine
Palmitoylcarnitine
Phenylalanine
Proline
Propionylcarnitine
Tyrosine
Valine
outlier #1
outlier #2
outlier #3
outlier #4
outlier #5
outlier #6
cases average
controls average

Heatmap of untargeted analysis features intensities generated using MetaboAnalyst 4.0

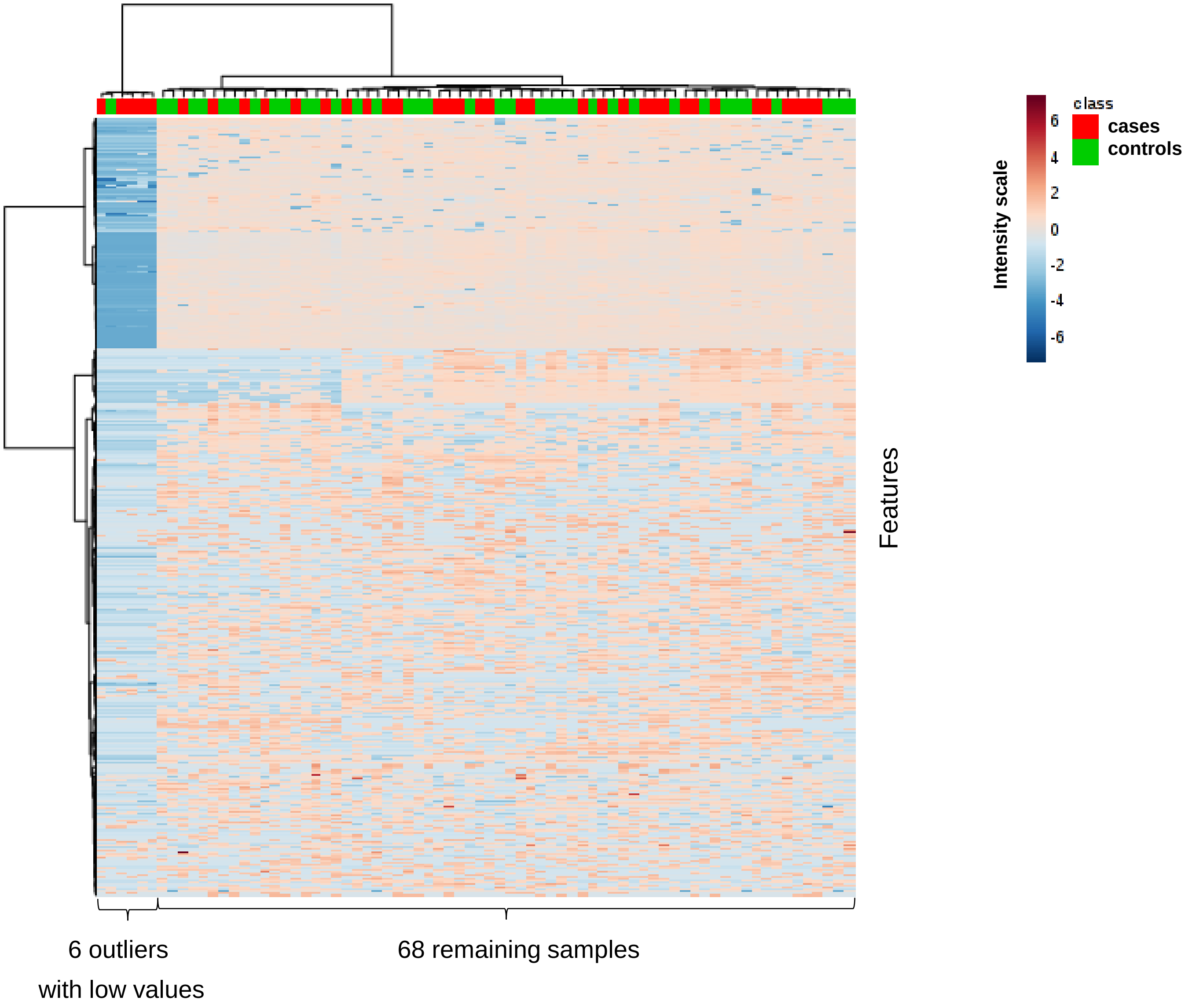

Supplement: Supplementary file 4 — Supplementary file4: Online Resource 4: Targeted analysis of outliers using TraceFinder (IS and unlabeled homologs) and heatmap of untargeted analysis. (PDF 599 KB) [file 12031_2020_1787_MOESM4_ESM.pdf]
