# Supplementary material for: Studying Autism Using Untargeted Metabolomics in Newborn Screening Samples
Source: J Mol Neurosci. 2021 Jan 30;71(7):1378–93. doi: 10.1007/s12031-020-01787-2 (PMC8233278; doi:10.1007/s12031-020-01787-2)
Supplement: Supplementary file 9 — Supplementary file9: Online Resource 9: Fragmentation profiles of the two unknown features to be monitored in future studies as well as methacholine as shown in Table 2 (PDF 174 KB) [file 12031_2020_1787_MOESM9_ESM.pdf]

Fragmentation profiles of the two unknown features to be monitored in future studies as well as methacholine as shown in Table 2.

The fragment masses and intensities were extracted from the aggregated .mgf file computed by MZmine and used in the network analysis.

ID8605 selected based on fold change	ID5593 selected based on significant p values before FDR correction	Methacholine selected based on significant p values before FDR correction
BEGIN IONS<br>FEATURE_ID=8605<br>PEPMASS=1014.4892<br>SCANS=8605<br>RTINSECONDS=398.449<br>CHARGE=1+<br>MSLEVEL=2<br>99.4840 5.0E3<br>130.2113 4.7E3<br>178.2334 6.2E3<br>498.1496 2.9E5<br>557.1633 3.9E5<br>616.1768 1.3E6<br>END IONS	BEGIN IONS<br>FEATURE_ID=5593<br>PEPMASS=231.1701<br>SCANS=5593<br>RTINSECONDS=166.747<br>CHARGE=1+<br>MSLEVEL=2<br>72.0808 4.2E4<br>84.9599 4.4E3<br>103.8799 2.1E3<br>144.7223 2.3E3<br>END IONS	BEGIN IONS<br>FEATURE_ID=159<br>PEPMASS=160.1332<br>SCANS=159<br>RTINSECONDS=26.97<br>CHARGE=1+<br>MSLEVEL=2<br>55.0546 2.1E5<br>59.0493 5.7E4<br>60.0810 6.1E5<br>101.0598 1.9E6<br>131.9749 2.3E4<br>END IONS
